# Use of youth-friendly health services and predictive factors: A community-based analytical cross-sectional study among young people in the Tamale metropolis

**DOI:** 10.1371/journal.pone.0314596

**Published:** 2024-12-30

**Authors:** Vivian Awuah, Gifty Apiung Aninanya, Benson Bionkum Konlan

**Affiliations:** 1 Department of Global and International Health, School of Public Health, University for Development Studies, Tamale, Ghana; 2 Department of Epidemiology, Biostatistics and Disease Control, School of Public Health, University for Development Studies, Tamale, Ghana; Far Eastern Memorial Hospital, TAIWAN

## Abstract

Globally, teenage pregnancies, unsafe abortions and sexually transmitted infections are on the increase among young people. However, their knowledge and uptake of youth-friendly sexual and reproductive health services are low. This study evaluated young people’s knowledge, attitude and utilization of youth-friendly health services in the Tamale Metropolis. 420 young people aged between 15 and 24 years were proportionately selected from four suburbs within the Tamale metropolis and a Likert scale was used to collect quantitative data. To determine the correlation between the relevant characteristics and attitudes toward youth-friendly health services, a logit model and chi-square statistics were applied. Respondents’ knowledge of youth-friendly health services was low (69%). A majority (71%) of them had a negative attitude towards youth-friendly health services and 63% of them had never used such services before. Level of education and religion were significantly associated with the knowledge levels of respondents while religion, employment status and individuals contributing to decision-making concerning the health of the participants were associated with attitudes towards the services. Cost and level of education were also associated with the uptake of youth-friendly health services. In conclusion, most young people had low knowledge, poor attitudes and low uptake of youth-friendly health services. Predictors of uptake of youth-friendly services were cost and educational level. The study suggests the need for Ghana Health Service to rigorously embark on sensitization programmes on the benefits of utilising youth-friendly health services and the cost of patronising these services could be subsidized to promote its utilization among the youth.

## Introduction

Youth refers to persons between the ages of 15 and 24 [1]. However, in Ghana, the youth are seen as people between the ages of 15 and 35 years [2]. This is a stage in life that results in changes in secondary sexual characteristics and eventually sexual and reproductive maturity [1, 3]. In 2019, the youth were about 1.2 billion of the global population and this figure may rise to 1.3 billion by 2030 [4]. The population of Ghana is youthful and approximately 6.9 million people in Ghana are adolescents, making up 22% of the country’s almost total population [5, 6].

Many young people transition to adulthood in good health, but others do not. Millions of these young people are forced into unwanted sex or marriage which makes them vulnerable to the risk of unwanted pregnancies, unsafe abortions, sexually transmitted infections (STIs) including HIV, and complications associated with labour and delivery. Nonetheless, these young people encounter challenges with access to SRH information [7, 8]. In some developing countries, only 10% of young people had used health services in the past twelve months and were informed about family planning [9]. Even though premarital sexual behaviours are on the rise among young people, only a small percentage of those who do not wish to become pregnant use a current method of family planning. Moreover, when they have access to sexual and reproductive health (SRH) information, adolescents are unable to utilise health services due to restrictive policies, long waiting hours, negative attitudes of health staff, and inadequate access to health services [7]. Every year, 21 million teenagers become pregnant worldwide, with Sub-Saharan Africa accounting for the majority. There were 140,000 new cases of HIV infection in 2022. Due to inadequate access to family planning services, HIV is increasing and unintended pregnancies are occurring [10]. Adolescent health has to be improved [11].

In Ghana, the health of the youth is also a public health issue [12]. Although the majority of young people are aware of family planning, only a small proportion of them use it [13], and this has led to poorer SRH outcomes [13]. Also, young people account for 20% of births, most of which are attributed to poor access to SRH information [14, 15].

Youth-friendly health services (YFHS) are designed to address the barriers faced by youth in accessing high-quality SRH [5, 16, 17]. Components of these services include behavioural counselling, family planning, and management of sexually transmitted diseases. SRH services for young people must provide a supportive environment, and improve SRH knowledge and services. YFHS must be accessible, acceptable, equitable, appropriate and effective [18, 19].

Any young person visiting the health facilities should be provided with respectful and confidential care. However, in developing countries, most young people do not use health services due to stigma, unfriendly health staff, and long waiting times, among others. In most cases in Ghana, these young people are confronted with impediments such as negative attitudes toward service providers, restrictive policies and unavailability of the service which affect their uptake of health services [19–21].

Although young people encounter diverse SRH challenges in low- and middle-income countries, evidence of their use of health services and their determinants is scanty [22]. Several systematic reviews of YFHS interventions found insufficient evidence to support the effectiveness of such interventions. In addition, uptake of YFHS is sub-optimal among young people. An earlier study among 690 adolescents in Ethiopia revealed that only 45% used health services and a mixed-method study in Ethiopia among over 800 young people revealed that about 64% of them had already utilized YFHS [23, 24]. Another study among 700 students in Ethiopia revealed that their uptake of YFHS was only 38.5% [24]. In Nigeria, most adolescents were aware of YFHS but uptake was very poor [25]. Prior evidence suggests that determinants of the use of health services were proximity, knowledge about the benefits of YFHS, drug availability, health services availability, treatment in a separate room and respect [24, 26].

In Ghana, evidence on the utilization of YFHS and associated factors is limited. Extensive work has been done on adolescent sexual and reproductive health [27–29] but not on the determinants of YFHS [21, 29]. A previous qualitative study in the West Gonja District found that knowledge and utilization of SRH services were low among adolescents [30] Another study among school adolescents in the Tamale metropolis revealed poor knowledge, attitudes, and practices toward SRH [31]. However, a recent quantitative study conducted among rural adolescents in the Kumbugu district in Northern Ghana revealed a moderate uptake of such services [21]. Presently, there is sketchy literature on the level of utilization of YFHS and its associated factors among Urban young people in northern Ghana. This study expands on previous literature to assess the knowledge, attitudes and utilization of YFHS among young people in the Tamale Metropolis.

## Methodology

### Study design

An analytical community-based cross-sectional study was carried out among conveniently selected young people [32]. The study design helped the authors to measure the outcome variable and the exposure variables at the same point in time [33] among young people in the Tamale metropolis from 13^th^ October to 13^th^ November 2021.

### Study setting

The study was conducted among conveniently selected young people in the Tamale Metropolis (Bupiela, Vittin, Tamale Central and Nyohini). Young boys and girls aged 14 to 24 years and living within the Tamale metropolis were considered eligible for the study. The metropolis is one of the 26 (twenty-six) administrative divisions that make up the Northern Region of Ghana. Tamale is made up of 267 settlements and it is divided into 4 sub-metro areas with a total population of 233,252; accounting for 9.4% of the region’s total population. Males make up 49.7% of the population, while females make up 50.3%. Almost thirty-seven per cent (36.5%) of the population are under the age of 15 [34]. There are four hospitals in the Tamale metropolis and more than five thousand nurses and midwives are found in the northern region that render services to clients.

### Sample size determination and sampling procedure

Using the formula of Yamane (1967), and considering a 5% margin of error, an estimated sample size of 399 young people was obtained. A total sample of 420 young people in the Tamale metropolis was eventually used to account for non-response [35]. A proportionate stratified sampling approach was initially designed, selecting 105 young people aged 15 to 24 from each suburb (Bupiela, Vittin, Tamale Central, and Nyohini) to ensure fair representation from all areas. However, within each suburb, young people were conveniently selected from various places, and questionnaires were distributed at exit points to respondents who were available to answer them.

### Data collection tools and procedures

A psychometric scale (Likert scale) was used to assess the “knowledge level”, “attitudes” and “utilization” of YFHS in the Metropolis. The scale contained four main sections; demographic characteristics of the respondents, knowledge of YFHS, attitudes towards YFHS and utilization of YFHS. Consequently, a seven-point agreement scale (1–3 = Low; 4–5 = Moderate; and 6–7 = High) was used to measure respondents’ agreement with a variety of statements to assess the “knowledge level”, and likewise for the “attitude” variable. The questionnaire was developed based on previous related literature and based on WHO guidelines on assessing factors associated with the utilization of YFHS [36–38]. The questionnaire was pretested among 25 adolescents in a suburb of the Tamale metropolis. Four trained postgraduate students of the University for Development Studies conducted face-to-face interviews among adolescents in English. Averagely each interview took about 45 minutes.

### Data processing and analysis

The Statistical Package for Social Sciences version 24 was used to analyse the data. Both descriptive and inferential analysis was done. A Chi-square test was performed to evaluate the associations between demographic characteristics and the utilization of YFHS. Also, Kendall’s coefficient of concordance (W) was used to determine which factors influenced their pursuit of YFHS, and by how much.

### Ethical consideration

Ethical approval was provided by the Kwame Nkrumah University of Science and Technology’s Committee on Human Research, Publication, and Ethics (CHRPE/AP/335/21). Also, an administrative approval letter was taken from the University for Development Studies and young people were duly informed about the rationale of the study.

All participants acknowledged knowing the elements of informed consent and consented to take part in the interview. Before the interviews were done, both parents and guardians gave their permission in writing for participants under 18 years old to participate in the study. The study’s aims, procedures, voluntariness, benefits and risks, compensation and confidentiality, as well as who to contact were all explained to the study’s underage participants’ parents and guardians to obtain their consent.

## Results

### Socio-demographic information

Four Hundred & Twenty participants (420) took part in the study which resulted in a 100% response rate. A little over half of the respondents (52%) were females, and the majority (52%) of them were between the ages of 14 and 19, with a Senior High School education (41%). Most respondents were Muslims (60%), 76% were single, and 15% of them were unemployed (Table 1).

**Table 1:** Socio-demographic information (n = 420).

Variable		Frequency	Percentage
Sex	Male	203	48.33
	Female	217	51.67
Age	14–19	217	51.67
	20–24	203	48.33
Education	Primary School	24.0	5.71
	Junior High School	75.0	17.86
	Senior High School	172	40.95
	Tertiary	144	34.29
	None	5.00	1.19
Religious	Christian	166	39.52
	Islam	254	60.48
	Traditionalist	0.00	0.00
Marital status	Single	321	76.43
	Married	74.0	17.62
	Divorced	13.0	3.10
	Widowed	5.00	1.19
	Co-habiting	7.00	1.67
Employment	Employed	108	25.71
	Unemployed	238	56.67
	Self-employed	74.0	17.62

### Knowledge of youth-friendly health service

**Fig 1:**
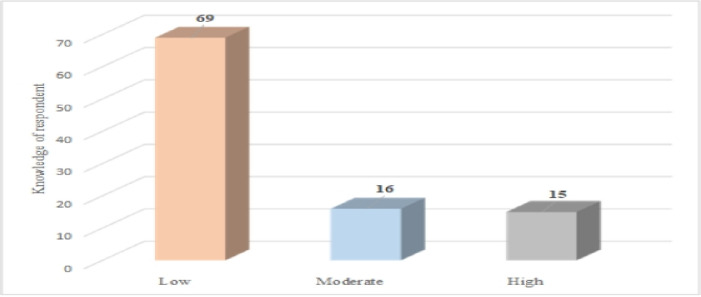
Knowledge of youth friendly health service.

The majority of respondents (69%) had little knowledge of YFHS. On the other hand, only 16% and 15% of the participants had moderate and high knowledge of YFHS respectively (Fig 1).

### Influence of demographic factors on knowledge of youth-friendly health service

Table 2 shows that there was no significant difference between sex and knowledge on YFHS (0.72), the level of association between age and knowledge was not significant (0.21), also there was no significant difference between marital status, employment status, and the knowledge young people have on youth-friendly health services with a p-value of (0.68). Also, education and religion with associated with the knowledge levels of young people on youth-friendly health services (Tables 2 and 3).

**Table 2:** Socio-demographics of respondents by knowledge score (N = 420).

Variables		Knowledge of YFHS (%)			Pooled	P-value
		Low	Medium	High		
Sex	Male	52.24	48.10	45.31	48.33	0.723
	Female	47.76	51.90	54.69	51.67	
	*	0.650				
Age	14–19	41.79	53.29	54.69	51.67	0.207
	20–24	58.21	46.71	45.31	48.33	
	*	3.154				
Education	Primary School	2.99	6.23	6.25	1.19	**0.000**
	Junior High School	40.30	14.19	10.94	5.71	
	Senior High School	38.81	43.25	32.81	17.86	
	Tertiary	17.91	34.95	48.44	40.95	
	None	0.00	1.38	1.56	1.19	
	*	35.294				
Religious	Christian	25.37	43.60	35.94	39.52	**0.019**
	Islam	74.63	56.40	64.06	60.48	
	Traditionalist	0.00	0.00	0.00	0.00	
	*	7.964				
Marital status	Single	76.12	76.12	78.13	76.43	0.682
	Married	17.91	18.34	14.06	14.06	
	Divorced	2.99	3.11	3.13	3.13	
	Widowed	1.49	1.38	0.00	0.00	
	Co-habiting	1.49	1.04	4.69	4.69	
	*	5.685				
Employment	Employed	17.91	25.61	34.38	25.71	0.308
	Unemployed	62.69	57.09	48.44	56.67	
	Self-employed	19.40	17.30	17.19	17.62	
	*	4.802				

*Pearson’s chi-square (*x*^2^) test was used to test the differences between the variables.

**Table 3:** Predictors of knowledge of respondents.

Model	Unstandardized Coefficients		Standardized Coefficients	t	Sig.
	B	Std. Error	Beta		
(Constant)	4.692	.567		8.278	**.000**
1. Age	-.302	.161	-.092	-1.876	.061
2. Sex	.201	.162	.061	1.241	.215
3. Religious denomination	-.058	.168	-.017	-.345	.730
4. Marital status	.024	.109	.011	.223	.823
5. Level of formal education	.255	.090	.143	2.823	**.005**
6. Employment status	-.182	.122	-.072	-1.489	.137

### Attitude towards youth friendly health services

**Fig 2:**
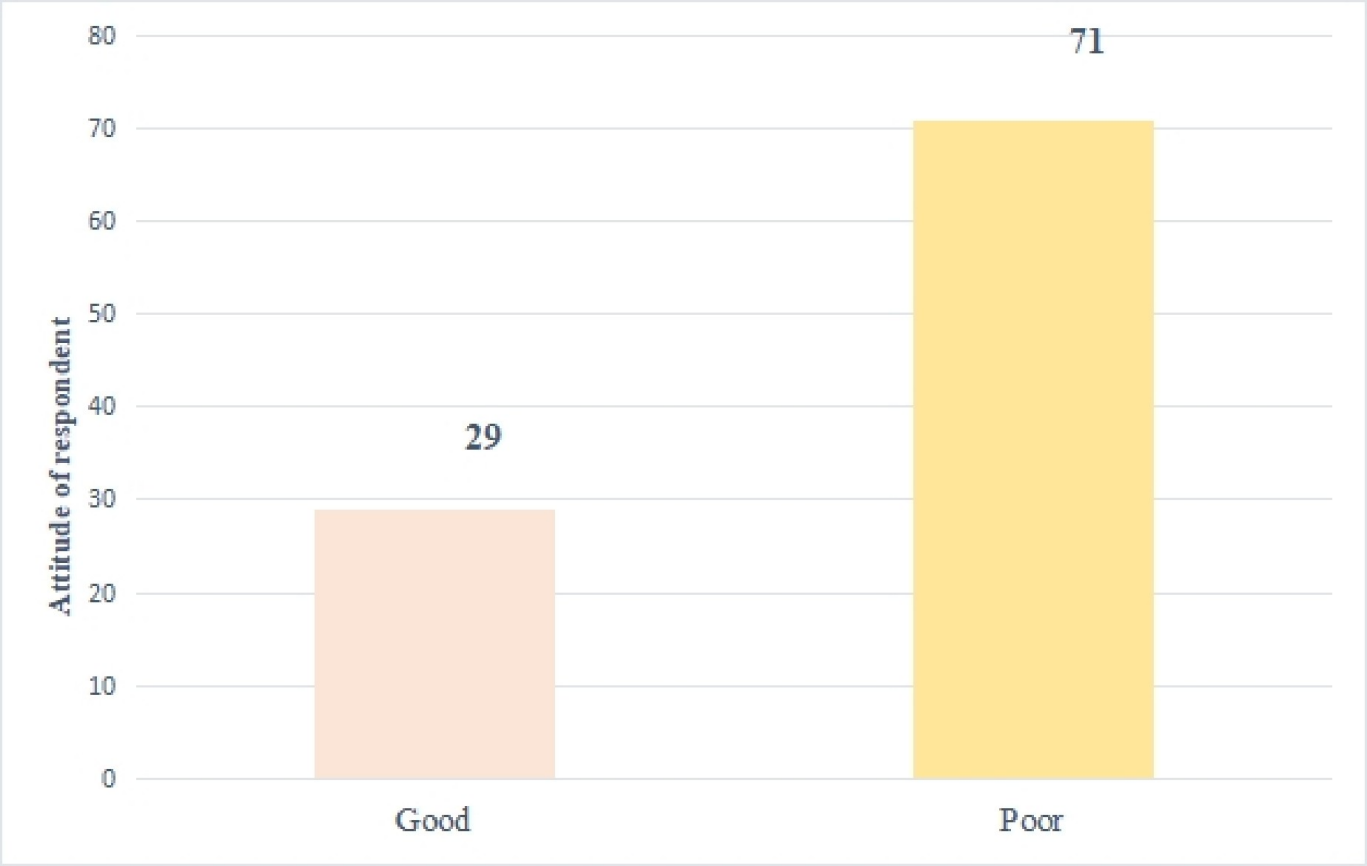
Attitude towards youth friendly health service.

The majority of respondents (71%) had a negative or low attitude toward youth-friendly health services (Fig 2).

### Influence of demographic factors on attitude towards youth-friendly health service

Table 4 shows no significant difference between sex and attitudes toward YFHS with a P-value of (0.74). The association between age and attitude was not significant with a P-value of (0.45) and there was no significant difference between religion, marital status, employment status, and the attitudes young people have towards youth-friendly health services with a p-value of (0.72), (0.24), and (0.24), respectively. But there was a significant difference (0.01) between education and the attitudes young people have towards youth-friendly health services.

**Table 4:** Socio-demographics of respondents by attitude score (N = 420).

Variables		Attitude on YFHS (%)			
		Good	Poor	P-value	Pooled
Sex	Male	49.59	47.81		48.33
	Female	50.41	52.19		
	*	0.111		0.739	
Age	14–19	48.78	52.86		51.67
	20–24	51.22	47.14		48.33
	*	0.580		0.446	
Education	Primary School	6.50	5.39		5.71
	Junior High School	18.70	17.51		17.86
	Senior High School	30.08	45.45		40.95
	Tertiary	44.72	29.97		34.29
	None	0.00	1.68		1.19
	*	12.868		**0.012**	
Religion	Christian	38.21	40.07		39.52
	Islam	61.79	59.93		60.48
	*	0.125		0.723	
Marital status	Single	82.11	74.07		76.43
	Married	15.45	18.52		17.62
	Divorced	1.63	3.70		3.10
	Widowed	0.81	1.35		1.19
	Co-habiting	0.00	2.36		1.67
	*	5.522		0.238	
Employment	Employed	30.08	23.91		25.71
	Unemployed	50.41	59.26		56.67
	Self-employed	19.51	16.84		17.62
	*	2.847		0.241	

*Pearson’s chi-square (*x*^2^) test was used to test the differences between the variables.

### Factors influencing attitudes towards youth-friendly health services

Table 5 below shows that the employment status of individuals contributes to decision-making concerning their use of youth-friendly health services. Sex, age, religious status, awareness of youth-friendly health services, promiscuity, availability of youth-friendly health services and the importance of YFHS to the young person was not significant factors. Also, individuals contributing to decision-making concerning the health of participants had a positive effect on respondents’ attitudes.

**Table 5:** Predictors of respondents’ attitudes toward YFHS.

Variables	Estimated result of logit model			
	Coefficient	Std error	P>|z|	Marginal effect
Sex	-0.0408	0.1321	0.757	-0.0138
Age	0.1027	0.1327	0.439	0.0348
Religious status	0.0672	0.1358	0.621	0.0227
Employment status	0.2278	0.1333	0.087	**0**.0777*
Awareness of YFHS	-0.2349	0.1953	0.229	-0.0755
YFHS are promiscuous	-0.1692	0.1457	0.245	-0.0564
Decision making	0.5255	0.1611	0.001	0.1639***
YFHS in your community	0.1787	0.1468	0.223	0.0616
Access YFHS before	0.2316	0.1396	0.097	0.0797*
YFHS important for adolescent	0.0817	0.1468	0.578	0.0274
Constant	-1.3582	0.3948	0.001	0.2837***
Number of Observations	420	LR chi2(10)	20.45	
Prob > chi2	0.0252	Pseudo R2	0.0403	
Log-likelihood	-243.7426			

### Utilization of youth-friendly health services

The majority of the respondents (68%) stated that they do not have YFHS but (63%) of them indicated that they have never used the services. But the few respondents who used the service disclosed that the facilities were welcoming and the service providers were friendly (Table 6).

**Table 6:** Utilization of youth-friendly health service.

Variables		Frequency	Percentage (%)
Presence of the YFHS facility	Yes	134	31.90
	No	286	68.10
Total		420	100
Ever used YFHS before	Yes	154	36.67
	No	266	63.33
Total		420	100
Facility environment welcoming	Yes	86	57.33
	No	64	42.67
Total		150*	100
Service Provider friendly	Yes	111	73.51
	No	40	26.49
Total		151*	100
Respond to concerns satisfactory	Yes	64	74.49
	No	24	25.51
Total		88*	100
Your information is kept confidential	Yes	75	84.10
	No	13	15.90
Total		88*	100
Important to access this information	Yes	290	69.05
	No	130	30.95
Total		420	100

*Represents frequencies which do not add up to 420 because respondents skipped questions

### Factors influencing utilization of youth-friendly health services

Table 7 shows the factors that influenced young people’s use of youth-friendly health services. Kendall’s ‘W’ of 0.402 indicates a 4% agreement among the respondents in the ranking of factors concerning the utilization of youth-friendly health services. Among the identified ranked factors, the cost of accessing youth-friendly health services, health problems or illness, health workers’ attitudes, distance to youth-friendly health services, and location of youth-friendly health services were the top five factors. Time (the waiting time), educational level, cultural belief, and religious reasons were the least factors influencing young people’s utilization of youth-friendly health services. Marital status was significantly associated with the uptake of YFHS (Table 8).

**Table 7:** Factors influencing utilization of YFHS among adolescents.

Variables	Mean	STD	Mean rank	Rank
Cost of accessing youth-friendly health services	1.23	1.07	4.56	1^st^
Health problems or illness	1.37	1.04	4.76	2^nd^
Health workers Attitude	1.38	1.03	4.82	3^rd^
Distance to youth-friendly health services	1.37	1.11	4.83	4^th^
Location of youth-friendly health services	1.42	1.12	4.95	5^th^
Time (the waiting time)	1.46	1.17	4.98	6^th^
Educational level	1.43	1.12	4.97	7^th^
Cultural belief	1.48	1.04	5.00	8^th^
Religious Reasons	1.92	1.21	6.14	9^th^
N	420			
Kendall’s W^a^	0.042			
Chi-Square	140.707			
P<0.05	0.000			

**Table 8:** Demographics and utilization of youth-friendly health service.

Model	Unstandardized Coefficients		Standardized Coefficients	t	Sig.
	B	Std. Error	Beta		
(Constant)	.422	.168		2.512	**.012**
1. Age	-.009	.048	-.009	-.178	.858
2. Sex	.017	.048	.017	.349	.727
3. Religious denomination	-.074	.050	-.075	-1.487	.138
4. Marital status	.071	.032	.110	2.193	**.029**
5. Highest level of formal education	-.012	.027	-.022	-.436	.663
6. Employment status	-.005	.036	-.006	-.130	.896

## Discussion

This study sought to determine young people’s knowledge, attitudes and uptake of YFHS within the Tamale metropolis. Generally, knowledge, attitudes and utilization of YFHS were poor. This is because young people in the area do not have accurate knowledge of SRH information as revealed by previous evidence [31]. Also, in this study, more than half of the respondents (69%) had poor knowledge of YFHS. This finding explains why respondents had poorer attitudes and sub-optimal uptake of YFHS among young people in the Tamale metropolis. This suggests that young people require rigorous sexual and reproductive health interventions to boost their knowledge levels, enhance their attitudes and improve their use of health services [20]. Similar findings were reported earlier in Ghana, where only 31% of respondents were aware of the existence of YFHS, and in Nigeria, where three-quarters (80%) of respondents were unaware of a specific YFHS serving their area [39, 40]. However, this result is higher than other studies in Nigeria which indicated that 52.7% of the respondents knew of the existence of YFHS [40]. However, it contradicts findings in an Ethiopian study where 78.5% knew numerous types of reproductive health [29] services. In Nepal, young people had higher SRH knowledge and practice. An earlier study reported that young people who knew about HIV/AIDS and voluntary counselling and testing were three times more likely to use YFHS than those who didn’t [41]. Therefore, higher knowledge and positive attitudes towards YFHS can undoubtedly inspire young ones to use health services. Also, nearly three-quarters of the respondents (71%) had a negative or poor attitude toward YFHS. This finding is consistent with another study that found that most young people who had RH disease in the three months before the study did not seek treatment because there were no available services, revealing their negative attitude toward SRH care and possibly linked to their perceptions of what is and is not acceptable. The current finding also contradicts a study conducted on the use of YFHS and related factors in Harare, Ethiopia, which revealed that the majority of the youth had a favourable attitude toward YFHS [23]. Similarly, Nalwadda *et al*. (2010) reported that attitudes of young people toward YFHS, particularly contraception, were influenced by their fear of being imprisoned [42].

This present study also revealed a significant relationship between attitude and education, implying that people who have received a form of education at YFHS will have a positive attitude toward it. In this study, there was no significant relationship between knowledge of YFHS and attitude, which is consistent with a study that found that most youths were unaware of YFHS despite its availability[40].

The present study also revealed that the majority of respondents (68%) do not have a YFHS, and 63% have never used a YFHS. Similar studies have also reported that the majority of young people (55.5%) did not use health services during the past year [26]. Equally, 62% of young people in secondary schools did not utilize youth-friendly health services [43]. In contrast, a study conducted in Harar, Ethiopia’s Eastern Region, found that 64% of young people had used YFHS [23]. However, in this study, few respondents (37%) had used YFHS and the majority of users identified cost as the most important factor that affects utilization of YFHS. The Ghana Health Service should collaborate with partners such as Marie Stopes International to establish youth-friendly corners and embark on health promotional programs on the strengths of using YFHS among young people in the Tamale Metropolis.

When compared to an earlier study, the cost was a major factor influencing young people’s use of YFHS [44]. Adolescent sexual and reproductive health services should be captured under the National Health Insurance Scheme [45–47]. Also, most young people are afraid that using YFHS will cause them to be judged harshly by adults in the community, making it difficult for them to seek financial support from their parents [39]. Religious reasons were the least identified component in this present study, indicating that young people will seek out youth-friendly health care regardless of their religion; this is in contrast to a report that claimed religion has a significant impact on how people feel about YFHS, students who attended religious services had a negative attitude toward YFHS [48]. However, a similar study found that young people do not want people in their community to see them visiting a YFHS centre because they are afraid of being stigmatized by their communities and religious groups because of their faith [49].

### Study limitations

The study has contributed to the literature on determinants of YFHS in most developing countries and has filled an important knowledge gap in the Tamale metropolis. Policymakers in Ghana in particular will learn a lot of lessons from the study to be able to shape policies on YFHS that will go a long way to contribute to the attainment of Sustainable Development Goal 3. However, in this study, participants were not randomly selected; a convenient sampling technique was used to select the participants. This implies the results may not be a true reflection of the situation among young people in the Tamale metropolis. Also, since this was a quantitative community-based cross-sectional design, conclusions of causal inferences could not be made. A qualitative study could also have provided information on the perceived factors that influenced the utilization of health services but this was not included in the study. Also, the sample size was not large enough due to logistical constraints. Future studies should randomly select all adolescents/young people in all the districts in northern Ghana and apply a mixed-method approach to rigorously assess the determinants of YFHS.

## Conclusions

The majority of young people (69%) had low knowledge of YFHS, about 71% of them had a negative attitude toward YFHS and the majority of them (63%) had never visited any YFHS centres. We would recommend the implementation of a targeted education campaign for the youth on the usefulness of YFHS that could improve knowledge, attitudes and uptake of YFHS among this target group in the Tamale Metropolis. Furthermore, utilization could also be enhanced by a youth subvention payment system, including the supply of free education material by the Metropolitan Assembly to make the services of YFHS accessible to the youth.

## Supporting information

S1 Data (SAV)
